# School Climate, Teacher-Child Closeness, and Low-Income Children’s Academic Skills in Kindergarten

**DOI:** 10.5539/jedp.v5n2p89

**Published:** 2015

**Authors:** Amy E. Lowenstein, Allison H. Friedman-Krauss, C. Cybele Raver, Stephanie M. Jones, Rachel A. Pess

**Affiliations:** 1 Fordham University, New York, USA; 2 Steinhardt School of Culture, Education, and Human Development, New York University, New York, USA; 3 Harvard Graduate School of Education, Massachusetts, USA; 4 Kean University, New Jersey, USA

**Keywords:** academic skills, adult support, kindergarten, low-income children, school climate, teacher-child closeness

## Abstract

In this study we used data on a sample of children in the Chicago Public Schools in areas of concentrated poverty-related disadvantage to examine associations between school climate and low-income children’s language/literacy and math skills during the transition to kindergarten. We also explored whether teacher-child closeness moderated these associations. Multilevel modeling analyses conducted using a sample of 242 children nested in 102 elementary schools revealed that low adult support in the school was significantly associated with children’s poorer language/literacy and math skills in kindergarten. Teacher-child closeness predicted children’s higher language/literacy and math scores and moderated the association between low adult support and children’s academic skills. Among children who were high on closeness with their teacher, those in schools with high levels of adult support showed stronger language/literacy and math skills. There were no significant associations between adult support and the academic skills of children with medium or low levels of teacher-child closeness. Results shed light on the importance of adult support at both school and classroom levels in promoting low-income children’s academic skills during the transition to kindergarten.

## 1. Introduction

A substantial body of research suggests that low-income children are at greater risk for poor academic skills than their higher-income peers (Jencks & Phillips, 1998; McLoyd, 1998; Ripke & Huston, 2006; Votruba-Drzal, 2006). As a key developmental context, schools hold the potential to attenuate the negative effects of poverty and promote low-income children’s academic success. Schools may exert their influence on children at multiple levels. Both school-wide processes, such as school climate (Esposito, 1999; Hoy & Hannum, 1997), and classroom-level processes, such as the teacher-child relationship (Hamre & Pianta, 2001; Pianta & Stuhlman, 2004) have been independently linked to students’ academic achievement. However, the research base on elementary school climate is quite thin. In addition, little is known about the effects of school climate during the transition to formal schooling, a major developmental milestone when children must learn to adapt to the academic, behavioral, and social demands of the kindergarten classroom. Finally, although we know that children’s classroom-level experiences are related to their success in school, we know very little about whether and how these experiences work in concert with school-level processes to influence children’s school performance. In an attempt to fill these gaps, the purposes of this paper were to explore associations between elementary school climate and low-income, racial/ethnic-minority children’s academic skills during the transition to kindergarten, and to examine whether and how teacher-child closeness moderates these relationships.

### 1.1 A Conceptual Framework for Understanding How Schools Influence Children

Bronfenbrenner’s ecological theory posits that human development is shaped by contextual influences (Bronfenbrenner, 1979). Of primary importance are the influence of microsystems, or the immediate settings in which development occurs, and proximal processes, or the pattern of activities, roles, and interpersonal relationships experienced by the developing person in a microsystem (Bronfenbrenner & Morris, 2006). Both school and classroom characteristics reflect microsystemic influences-proximal processes occur in the children’s classroom, as well as in other settings throughout the school. Bronfenbrenner further posits that human development is influenced by mesosystems, or the interactions between these microsystems. For instance, the negative effects of attending a school with a high student-teacher ratio could be exacerbated by an unsupportive teacher-child relationship.

Risk and resilience theory posits that children faced with significant adversity can succeed in the presence of protective factors, which reduce the negative effects of risk (Luthar & Cicchetti, 2000; Masten & Gewirtz, 2006). Risk and protective factors include both individual-level characteristics, such as temperament, and relational and/or contextual characteristics, such as the nature of the parent-child relationship. Literature on the cumulative effects of risk further suggests that the sheer number of risk factors, including family and social factors, increases the likelihood that children’s development will be compromised (Appleyard, Egeland, van Dulmen, & Sroufe, 2005; Evans, 2004; Sameroff, Seifer, Baldwin, & Baldwin, 1993). The inverse is also true: exposure to more protective factors benefits the cognitive and social-emotional development of children exposed to risk (Seifer, Sameroff, Baldwin, & Baldwin, 1992).

Taken together, ecological theory and risk and resilience theory suggest that children are influenced by the environments to which they are exposed. Furthermore, features of these environments can serve as either risk or protection for low-income children, who begin their school careers at risk for poor academic and developmental outcomes. Finally, these risk and protective factors may interact to either protect children from or place them at increased risk for poor developmental outcomes.

### 1.2 Poverty, School Climate, and Children’s Academic Performance

It is now widely accepted that poverty predicts children’s higher risk of poor academic skills, even after accounting for parent and family characteristics. Children from poor families are more likely to have difficulties with school readiness and academic performance and are at least twice as likely to be held back in school as their higher-income peers (McLoyd, 1998; Reardon, 2011; Ripke & Huston, 2006). Exposure to poverty in early childhood is associated with poor academic performance in both the short and long term, with increasing effects that may set children on trajectories that widen the income gap in achievement over time (Duncan & Brooks-Gunn, 2000; Votruba-Drzal, 2006).

One way that poverty may have its negative impact on children’s opportunities for learning is through low-income children’s higher likelihood of enrollment in schools with more negative school climate. School climate is defined as the “personality” or “character” of school life (Anderson, 1982; Cohen, McCabe, Michelli, & Pickeral, 2009). It includes the norms, beliefs, and expectations held by teachers, students, and other school staff, as well as the quality and consistency of interpersonal relationships within the school (Brookover et al., 1978; Haynes, Emmons, & Ben-Avie, 1997). Traditionally, measures of school climate have relied on aggregates of individual students’ or teachers’ perceptions of the school environment.

A growing body of research on school-based interventions, many of which target school climate with the goal of improving children’s academic and social-emotional skills, provides suggestive evidence for the causal effects of school climate on children’s academic achievement. For example, the Child Development Project provides a comprehensive, whole-school approach to promoting prosocial behavior in elementary schools by building community in the classroom, across grades, school-wide, and with families. Experimental and quasi-experimental evaluations of the program suggest that it has positive effects on children’s motivation to learn; interpersonal relationships; social attitudes, values, and skills; behavior; and academic achievement in the short and long term (Battistich, 2008; Clayton, Ballif-Spanvill, & Hunsaker, 2001; Solomon, Battistich, Watson, Schaps, & Lewis, 2000). Although these results indicate that universal school-based interventions can benefit children’s school performance, the comprehensive nature of the program makes it difficult to disentangle the effects of specific program components and distinguish between the classroom- and school-level processes underlying its impacts. This work points to the need for a better understanding of the mechanisms linking children’s school experiences to their academic skills.

A larger number of correlational studies have yielded support for the role of school climate in predicting older students’ academic and behavioral outcomes (Hoy & Hannum, 1997). Although both the terminology for and operationalization of school climate constructs tend to vary across studies, a number of themes have emerged from this body of work that highlight the importance of school climate to students’ academic performance. In brief, this past research suggests that student and teacher reports of *school safety* and *a climate characterized by adult support* are each related to students’ academic achievement in elementary, middle, and/or high school. We review the evidence for each of these dimensions below.

#### 1.2.1 School Safety

Studies of school safety typically examine the prevalence of bullying, gang violence, and/or violence within schools and its association with student outcomes. Although more attention has been paid to links between school safety and students’ social-emotional functioning (see, for example, Flannery, Wester, & Singer, 2004; Gottfredson, Gottfredson, Payne, & Gottfredson, 2005; Janosz et al., 2008), there is evidence that school safety is also related to students’ academic achievement. Teachers’ ratings of school safety have been linked to schools’ mean math and reading achievement scores in both elementary and middle school and to residual gains in aggregated math and reading scores in elementary school (Gottfredson & Gottfredson, 1989). Other research confirms the link between school safety, as measured by administrative reports, and aggregated math and reading test scores in middle school (Gronna & Chin-Chance, 1999).

Related research on bullying has found that a higher prevalence of teasing and bullying in high school is predictive of higher school dropout rates (Cornell, Gregory, Huang, & Fan, 2013) and that elementary school children in 3^rd^ through 5^th^ grades who were directly involved in bullying as a victim, bully, or both were more likely to display poor school achievement than bystanders (Glew, Fan, Katon, Rivara, & Kernic, 2005). Together, these findings suggest that being in an unsafe school may have serious repercussions for children’s academic progress, although none of this research included children as young as kindergarten age.

#### 1.2.2 A Climate Characterized by Adult Support

A second school-level construct that has been identified as important for learning is adult support for students’ academic success. Several studies have examined the degree to which the teachers at a school show commitment to their students and the school (e.g., by devoting extra time and effort to motivating and nurturing students). These studies have found that measures of teacher commitment are strong predictors of students’ school performance in both elementary and middle school, especially among racial/ethnic-minority students (Brookover et al., 1978; Esposito, 1999; Hoy & Hannum, 1997; Johnson, Livingston, Schwartz, & Slate, 2000). These results suggest that the degree to which students feel supported by the teachers in their school is strongly related to their school success. Although research on the importance of students’ school connectedness has highlighted the role of support from a broader group of adults in the school (Cohen et al., 2009; McNeely, Nonnemaker, & Blum, 2002), most research on adult support has focused exclusively on teachers. In the current paper, we extend this work by capturing the support of both teachers and other adults in the school in our measure of adult support.

### 1.3 Classroom Processes: Teacher-Student Relationships and Children’s Academic Skills

One important distinction to be made in research on children’s educational experiences is between children’s experiences in schools and classrooms. Simply put, a child might view his or her school relatively negatively overall but may have extensive opportunities for building a positive relationship with his or her teacher within the safety of the classroom. In an attempt to understand the extent to which children’s adaptation to early social environments in school is related to their school success, a separate body of research has examined key characteristics of the teacher-student relationship. This work suggests that children’s abilities to form warm, trusting, and low-conflict relationships with teachers in the early elementary school years facilitate their social adaptation to school and, in turn, their academic success (Birch & Ladd, 1997; Entwisle & Hayduk, 1988; Pianta, 1999; Pianta, Steinberg, & Rollins, 1995). A number of possible pathways have been proposed to explain these associations, including one in which a warm, affective connection with an adult facilitates children’s positive affect, engagement, and attitudes toward school. In addition, a close teacher-child relationship offers children a secure base from which to explore the school environment and may facilitate high teacher expectations for student performance, thereby promoting student achievement early on (Birch & Ladd, 1997; Entwisle & Hayduk, 1988; Hamre & Pianta, 2001).

Based on the work of Pianta (1992), the nature of the teacher-student relationship is typically measured using teacher ratings of three qualitatively distinct dimensions: closeness, conflict, and dependency. Although the quality of the teacher-student relationship has generally been found to be a stronger predictor of children’s behavioral than academic outcomes, elementary-school teachers’ reports of higher levels of closeness and lower levels of conflict and dependency in the teacher-child relationship are also predictive of students’ concurrent and subsequent academic performance, especially among children at risk for academic problems (Birch & Ladd, 1997; Burchinal, Peisner-Feinberg, Pianta, & Howes, 2002; Hamre & Pianta, 2001; Pianta & Stuhlman, 2004).

### 1.4 Joint Influences of School and Classroom Characteristics on Child Outcomes

Exposure to social risk across different contexts (family, school, neighborhood) has been linked to students’ poorer academic achievement in elementary school and during the transition to middle school (Burchinal, Roberts, Zeisel, & Rowley, 2008; Morales & Guerra, 2006). However, less is known about the joint influence of risk and protective factors within schools or during the transition to elementary school. Despite evidence that social and relational aspects of both the school and classroom environments are related to students’ academic performance, we know very little about whether and how these two environments interact. Overlap between these two social contexts—schools and classrooms—(e.g., the importance of teachers and teacher-student interactions to each) suggests that they may interact in important ways to either support or constrain children’s academic growth. For example, if the quality of the teacher-student relationship is positively related to both children’s school adjustment and their academic performance, might it serve a protective role for students exposed to negative school climate?

With few exceptions (see Konishi, Hymel, Zumbo, & Li, 2010), this research question is relatively unexplored. Prior work that considers both school- and classroom-level factors suggests that students’ ratings of school climate vary by their perceptions of teachers’ interpersonal skills and whether their teachers really care about them, as well as by classroom-level factors such as class size (Hallinan, Kubitschek, & Liu, 2009; Koth, Bradshaw, & Leaf, 2008). However, this work does not link these features of children’s school and classroom experiences to child outcomes. Given that teacher- and classroom-level characteristics appear to be intertwined with school climate, we might expect to see significant interactions between the teacher-student relationship and school climate when predicting children’s academic skills.

### 1.5 The Present Study

In the review presented above, we highlighted several empirical gaps in our understanding of the relationship between elementary school climate and children’s academic skills in low-income communities. Furthermore, we know very little about the role of school climate as younger children transition to formal schooling, enter kindergarten, and face the complex task of adapting to the demands of the elementary school classroom and school setting. Finally, although we know that the teacher-student relationship is linked to children’s school success and can play a protective role for children from low-income families, little is known about the role it serves in schools of varying quality. To address these important and relatively unexplored research questions, the purposes of the current paper are to: (1) explore associations between elementary school climate and low-income children’s language/literacy and math skills during the transition to kindergarten, and (2) examine the teacher-child relationship as a moderator of these associations.

We capitalize on a subsample of young, racial/ethnic-minority children who made the transition from federally funded Head Start programs to public-school kindergartens as part of a larger longitudinal intervention study conducted in low-income communities in Chicago. Importantly, we were able to include these children’s pre-academic school-readiness scores prior to their entry into kindergarten, as well as demographic characteristics, as a means of statistically controlling for ways that children may have “sorted” into schools of higher versus lower quality, thereby reducing the threat of selection bias.

## 2. Method

### 2.1 Sample

Data for this paper come from the Chicago School Readiness Project (CSRP), a multi-component, cluster-randomized efficacy trial implemented in 35 Head Start-funded classrooms. The CSRP was designed to support low-income children’s self-regulation through the provision of extensive training and support to teachers in how to effectively manage children’s dysregulated behavior (see Raver et al., 2009). The 35 Head Start classrooms were located in 18 Head Start sites in seven high-poverty neighborhoods in Chicago. These 18 sites were randomly assigned to either a control group or an intervention group. Of the 602 3- to 5-year-old children enrolled in these 35 classrooms at baseline (year “T”, the preschool year—either the 2004–2005 or 2005–2006 school year), 338 were eligible for kindergarten in fall of the following school year (i.e., 5 years old by September 1^st^ in the year in which the CSRP team conducted a “T+1” follow-up data collection effort—either the 2005–2006 or 2006–2007 school year). (^Note 1^) An additional 15 children enrolled in kindergarten despite the fact that they were not yet 5, making a total of 353 children who could have entered kindergarten in fall of T+1. Of these 353 children, 246 were enrolled in kindergarten at a Chicago public school at T+1. Four of these children did not have data on either of the academic-outcome measures. Thus, the sample of children included in the current paper consists of the 242 children who were in kindergarten in the year following the Head Start year in one of 102 Chicago Public Schools (CPS), for whom complete language/literacy and math data were collected in winter of the T+1 kindergarten year.

There were a number of reasons why the remaining children who were eligible for kindergarten were excluded from our sample, including that they spent an additional year in preschool, entered kindergarten outside of CPS, entered special education programs, or did not provide information on their whereabouts in the year after baseline. There were no significant differences in gender, race/ethnicity, or family income-to-needs at baseline between the 242 children in the current analytic sample and the 111 age-eligible children who were excluded from the sample.

Approximately 62% of the current analytic sample was African American, 30% was Hispanic, and 8% was white/Non-Hispanic or biracial. About 52% of the sample was male. On average, children were 5.25 years of age when their language/literacy and math skills were assessed and came from families with an income-to-needs ratio of 0.69, as measured when they were in Head Start. The income-to-needs ratio was computed by dividing annual household income, adjusted for family size and composition, by the U.S. Census poverty threshold for the current year. An average income-to-needs ratio of 0.69 indicates that children in the current sample came from families who were, on average, living below (at 69% percent of) the federal poverty threshold. On average, there were 2.37 CSRP children in each CPS school in the sample, but the number of children per school ranged from 1 to 19. Among the 102 CPS schools in which the 242 children were nested, the average total enrollment was approximately 743 students. There was substantial variation around this mean, with a standard deviation of 684 and a range of 157–6,306. These schools served very large proportions of disadvantaged students at risk of school failure. For example, in the average school to which a CSRP-enrolled child transitioned, 89% of students were from low-income families (defined as being eligible for free- or reduced-price lunch) (range: 12–100%), only 65% of students met or exceeded Illinois state reading and math standards (range: 35–98%), and 15% of students were English Language Learners (ELLs) (range: 0–55%).

Aside from their age, there were no differences between children in the full CSRP sample at baseline and those in the current analytic sample on gender, race/ethnicity, or family income-to-needs. The subsample used in the present analysis offers a valuable empirical snapshot of the experiences of low-income children in the West and South sides of Chicago, as the current subsample was similar in many ways to the larger community of children attending CPS kindergartens at that time. For example, 86% of the study subsample and 83% of CPS kindergartners were eligible for free or reduced-price lunch (Chicago Public Schools, 2012). Although the proportion of racial/ethnic minorities was similar across the study subsample and the population of CPS kindergartners (95% and 91%, respectively), African-American children were slightly over-represented in the study subsample (62%) compared to the broader population of CPS kindergartners (42%), while Hispanic children were slightly under-represented, representing 30% of the study subsample and 45% of the broader CPS kindergarten population (Chicago Public Schools, 2012).

### 2.2 Measures

#### 2.2.1 Children’s Academic Skills

Teachers reported on children’s language and literacy and mathematical thinking skills using the kindergarten version of the Language, Literacy, and Mathematical Thinking Measure (LLM; U.S. Department of Education, 2002) in winter of the kindergarten year. The LLM is a modified version of the Academic Rating Scale (ARS; U.S. Department of Education, 2002), which indirectly assesses the processes and products of children’s learning in school. Teachers were asked to compare the target child to same-age peers. CSRP used a slightly modified version of the LLM that contained 20 items, each on a 1-to-5 Likert scale. The two subscales of the LLM, Language and Literacy and Mathematical Thinking, reflect the averages of 12 and 8 items, respectively. Example items reflecting language/literacy are: “Produces rhyming words” and “Reads simple books independently”. Example items reflecting math are: “Sorts, classifies, and compares math materials by various rules and attributes” and “Shows an understanding of the relationship between quantities”. Cronbach’s alphas for the full sample of CSRP children were 0.95 for both the language/literacy and math subscales.

To assess the validity of these teacher ratings of children’s academic skills, we examined correlations between LLM language/literacy scores and DIBELS (Dynamic Indicators of Basic Early Literacy Skills) scores for the subset of children in our sample for whom they were available in kindergarten (*n* = 121). The DIBELS is an early reading diagnostic and formative assessment that measures progress on a set of five foundational literacy skills. CPS administers the DIBELS in grades K-2. The LLM language and literacy scale was moderately and significantly correlated with the four DIBELS scales that were available in spring of the kindergarten year (Letter Naming Fluency, Phoneme Segmentation Fluency, Nonsense Word Fluency, and Word Use Fluency), with correlations ranging from 0.30 to 0.54. These associations lend credibility to the LLM as a valid measure of children’s language and literacy skills. A comparable assessment of children’s math skills was not available.

In spring of the Head Start year, students’ pre-academic skills were assessed using three subscales from the National Reporting System (NRS; U.S. Department of Health and Human Services, 2003), a cognitively oriented, federally mandated assessment of Head Start preschoolers’ letter-naming, vocabulary, and early math skills. Data were collected by a multiracial group of master’s level assessors who had been extensively trained and certified in direct assessment procedures. The letter-naming score captures the proportion of letters correctly identified (Zill, 2003a). A shortened version of the Peabody Picture Vocabulary Test (PPVT-III; Dunn & Dunn, 1997) was used to measure children’s receptive vocabulary skills. The early math skills score reflects children’s knowledge of basic addition and subtraction (Zill, 2003b).

#### 2.2.2 Student-Teacher Relationship

Teachers reported on their relationship with the target child in winter of the kindergarten year using the Student-Teacher Relationship Scale (STRS; Pianta, 2001). The STRS is used to capture student-teacher relationship patterns in terms of conflict, closeness, and dependency with children in preschool through grade 3. It has 28-items and uses a 5-point Likert scale to assess a teacher’s perceptions of his or her relationship with a particular child, the child’s interactive behavior with the teacher, and the teacher’s beliefs about the child’s feelings toward the teacher. The CSRP version of the STRS contains only the 15 items that comprise the conflict and closeness subscales. Only the eight items used to measure closeness were included in the current paper; the closeness score was the sum of these items (Cronbach’s alpha was 0.86 for the full sample of CSRP children). Example items include: “I share an affectionate, warm relationship with this child”, and “If upset, this child will seek comfort from me”.

#### 2.2.3 Child-Level Covariates

The child’s primary caregiver completed a survey on the child’s background and demographic characteristics in fall of the Head Start year (at time T) and fall of the kindergarten year (at time T+1). Measures taken from this survey and other sources and included as covariates in the current analysis were: (a) child membership in the racial/ethnic category of African American versus Hispanic/other, (b) child gender, (c) child age at the time of assessment, (d) the family’s income-to-needs ratio from the Head Start year, (e) whether or not the child’s home language was Spanish, (f) direct assessments of the child’s pre-academic skills in spring of the Head Start year (letter naming and receptive vocabulary skills when predicting language and literacy skills; early math skills when predicting math skills), (g) whether the child had been in a Head Start site that was assigned to the CSRP treatment condition (versus the control condition), and (h) a set of site-pair dummy variables that capture which of 9 pairs of CSRP Head Start sites the child had been in (sites were matched on 14 different characteristics before being randomly assigned to treatment and control groups). (^Note 2^) In addition, because a substantial share (43%) of children in our sample had teachers whose race/ethnicity differed from their own, and given evidence that a racial/ethnic mismatch between teacher and student is associated with poorer student-teacher relations and student academic outcomes (Dee, 2004; Zimmerman, Khoury, Vega, Gil, & Warheit, 1995), we included (i) a covariate for whether there was a racial/ethnic match between teacher and child. By accounting for variation in children’s academic skills that was associated with a teacher-child racial/ethnic match, we were also able to increase the precision of our estimates of the relationship between teacher-child closeness and children’s academic skills.

#### 2.2.4 School Climate and School-Level Covariates

School climate was measured using two aggregates that were created using items from the Student Connection Survey (SCS) and the CPS CEO Report. The SCS was developed in association with the Collaborative for Academic, Social and Emotional Learning (CASEL), the American Institutes for Research (AIR), and the Consortium of Chicago School Research (CCSR). It is based on the Conditions for Learning Survey, which measures school climate in the elementary- and high-school grades (CASEL, 2009; Osher et al., 2008; Osher, Kendziora, & Chinen, 2008; Osher, Sidana, & Kelly, 2008). The SCS was administered to all sixth-, seventh-, and eighth-grade CPS students starting in the spring of the 2008 school year. Since 80% of CPS elementary schools serve either kindergarten or prekindergarten through eighth grade, these data are reflective of CPS elementary schools. The survey records students’ feelings about their school’s physical and emotional safety, academic rigor, and student support, and about students’ social and emotional learning and participation in extracurricular activities. It contains 153 items for sixth- and seventh-graders and 171 items for eighth-graders. CPS provided us with 36 items aggregated to the school level, 20 of which were used to create two measures of school climate: unsafe climate and low adult support. Examples of items reflecting unsafe climate are: “How safe do you feel in your classes”? which captures the percentage of students who rated their school as somewhat safe or not safe, and “I worry about crime and violence in school”, which captures the percentage of students who indicated that they agreed or strongly agreed with this statement. Examples of items reflecting low adult support are: “Adults in this school are usually willing to make the time to give students extra help”, and “Teachers help me make up work after an excused absence”, each of which captures the percentage of students who indicated that they disagreed or strongly disagreed with the statement.

The CPS CEO Report (Chicago Public Schools, 2008) provides a summary of various characteristics of each of the Chicago Public Schools, including general school information (e.g., total school enrollment), information on whether the school made Adequate Yearly Progress (AYP) on No Child Left Behind accountability standards, and the percentage of students who met or exceeded state standards for math and reading on the Illinois Standards Achievement Test (ISAT). Two items from the CEO Report (school attendance rate and school mobility rate) were included in the unsafe climate aggregate and four items were used as school-level covariates (school enrollment and the percentages of students in the school who were from low-income families, who were English language learners, and who met or exceeded state standards for math and reading). (^Note 3^) All items were from 2008, to correspond with the year in which the SCS data were collected.

In total, the unsafe climate aggregate was based on 15 variables and the low adult support aggregate was based on 7 variables. To create the aggregates, the constituent items were converted to z-scores and then averaged. Prior measurement work involving exploratory and confirmatory factor analysis indicated that these two school climate factors provided a good fit to the data (Lowenstein, Raver, Jones, & Pess, 2011). Cronbach’s alphas for the aggregates included in the present analysis were 0.96 for unsafe climate and 0.81 for low adult support. The aggregates were not significantly correlated with each other (*r* = 0.02). Because information on school climate was reported by middle-school students and some items were less directly relevant to the daily activities of elementary school children than others, we use these two aggregates as rough proxies for the “temperature” of the elementary schools into which CSRP children transitioned at the start of the kindergarten year.

In trying to capture empirically accurate measures of school quality, this study faced a challenge in temporal sequence: while CSRP children were in kindergarten in either the 2005–2006 or 2006–2007 school year, the SCS survey was first administrated a short time later, in the 2007–2008 school year. Prior work using a nationally representative sample of schools from the ECLS-K suggests a high level of stability in school context between kindergarten and third grade (Lowenstein et al., 2015). Additional analyses using the current sample (not shown) also suggest very high levels of stability in school-level indicators of quality, such as the percentage of students with proficiency in language arts and mathematics, as indicated by 3^rd^-grade standardized test scores. In light of the high likelihood that other indicators of school quality, such as safety and support characteristics, were slow to change from year to year, we used the 2008 school-climate data as a robust (if not perfect) proxy for school climate in both 2006 and 2007.

### 2.3 Analytic Approach

To address our research question about the role of school climate in predicting children’s academic skills in kindergarten, we used multilevel modeling to account for the fact that children were nested in elementary schools (using Mplus version 6.12).

Multilevel modeling allows for the simultaneous estimation of variance associated with individual (within-participants) and population (between-participants) change based on the specification of fixed- and random-effect variables in the model (Raudenbush & Bryk, 2002). With these data it was possible to estimate associations between school-level variables (e.g., dimensions of school climate) and children’s academic skills, net of person-level (e.g., demographic and ecological characteristics of children and families) and school-level (e.g., school size, percentage of low-income students in the school) characteristics.

Associations between school climate and children’s academic skills (language/literacy and math) in kindergarten were modeled with two equations, with the level-1 (child-level) equation specified as follows:

(1) $$Y_{\text{ij}}=\beta_{0j}+\sum_{m}\beta_{j}X_{\text{ij}}+r_{\text{ij}}$$

where Y_ij_ is the academic skill score of child i in school j and Σ*_m_*β_j_X_ij_ represents the sum of *m* child and family characteristics, such as child race/ethnicity, family income-to-needs ratio, and the closeness of the teacher-child relationship in kindergarten. r_ij_ is a random error term. We used auto-lagged models in which we controlled for children’s pre-academic abilities during the Head Start year. Including controls for children’s baseline academic abilities (language and math) made our models more rigorous and conservatively specified, allowing for greater precision in our estimates.

Correspondingly, the level-2 (school-level) equation was specified as follows:

(2) $$\beta_{0j}=\gamma_{00}+\gamma_{01}C_{1j}+\gamma_{02}C_{2j}+\sum_{p}\gamma_{j}S_{j}+u_{0j}$$

where C_1j_ and C_2j_ are two school-climate aggregates (estimated independently) and Σ*_p_*γ_j_S_j_ represents the sum of *p* school-level characteristics, such as school size and the percentage of students in the school who met or exceeded IL state reading and math standards.

We first estimated associations between school climate and children’s academic (language/literacy and math) skills during the transition to kindergarten. Then, we investigated whether the relationship between school climate (unsafe climate and low adult support) and children’s academic skills was moderated by teachers’ reports of teacher-child closeness by including cross-level interactions between teacher-child closeness and each of the school climate aggregates.

Missing data were handled using Full-Information Maximum Likelihood (FIML) estimation. At the school level, between 2.9% and 19.6% of schools were missing data on each of the school-level variables. At the child level, 3% of cases were missing data on closeness with the teacher in kindergarten, 10% were missing data on family income-to-needs, 5% were missing information on child age at the time of the teachers’ rating of children’s academic skills, 10% were missing information on whether there was a teacher-child racial/ethnic match, and 13% were missing data on pre-academic skills in Head Start.

## 3. Results

### 3.1 Descriptive Statistics

Table 1 shows descriptive statistics for the level-1 and level-2 variables used in the analyses and Table 2 shows correlations among the level-1 variables. Standardized versions of the measures of unsafe climate and low adult support are shown in Table 1. Raw means were 38.57 for unsafe climate and 45.88 for low adult support, which roughly capture the percentages of middle-school students in the school who endorsed statements about their school being unsafe (unsafe climate) and the adults in their school being unsupportive (low adult support). These mean scores mask substantial variation, with scores for unsafe climate ranging from 16.94 to 54.19 and those for low adult support ranging from 10.71 to 56.71. As shown in Table 2, there were low-to-moderate significant correlations among the three measures of children’s pre-academic skills in Head Start, ranging from 0.27 to 0.58. The measures of children’s language/literacy and math skills in kindergarten were significantly and highly correlated with each other (*r* = 0.88).

### 3.2 Multilevel Models Examining Predictors of Children’s Academic Skills in Kindergarten

We used multilevel modeling to examine the associations between elementary school climate (unsafe climate and low adult support) and children’s academic skills during the transition to kindergarten. Results from 2-level unconditional multilevel models suggested that a significant portion of the variance in children’s academic skills was attributable to between-school differences (ICCs: 0.30 for language/literacy, 0.33 for math). Table 3 shows the results of the 2-level analyses that examined child- and school-level predictors of children’s teacher-reported language/literacy and math skills during the transition to kindergarten. Model 1 includes only the child- and school-level predictors; model 2 includes interactions between the two school climate aggregates and teacher-reported closeness with the child.

#### 3.2.1 Language and Literacy Skills

As shown in Language and Literacy Model 1 in Table 3, children enrolled in schools that were independently characterized as having low adult support were rated by their teachers as having poorer language/literacy skills in kindergarten, even after controlling for their earlier language/literacy skills in Head Start (*B* = −0.16, S.E. = 0.08, *p* = .047). Two school-level covariates were also significantly associated with children’s language and literacy skills. Children in schools with larger total enrollments and larger percentages of English Language Learners (ELLs) were rated by teachers as showing poorer language/literacy skills, net of their language/literacy skills in Head Start (*B* = −0.02, S.E. = 0.01, *p* = .02, and *B* = −0.02, S.E. = 0.01, *p* = .02, respectively).

Children whose teachers reported higher levels of closeness with them were also rated by teachers as showing stronger language/literacy skills in kindergarten (*B* = 0.04, S.E. = 0.01, *p* < .001). It is important to note that teacher-child closeness was a significant predictor of children’s language and literacy scores in kindergarten even after taking into account considerable stability in children’s language and literacy scores between Head Start and kindergarten (i.e., children who scored higher on the letter naming and receptive vocabulary subscales of the NRS were rated by teachers as showing stronger language/literacy skills in kindergarten, *B* = 0.77, S.E. = 0.19, *p* < .001, and *B* = 0.86, S.E. = 0.41, *p* = .04, respectively).

**Figure 1:**
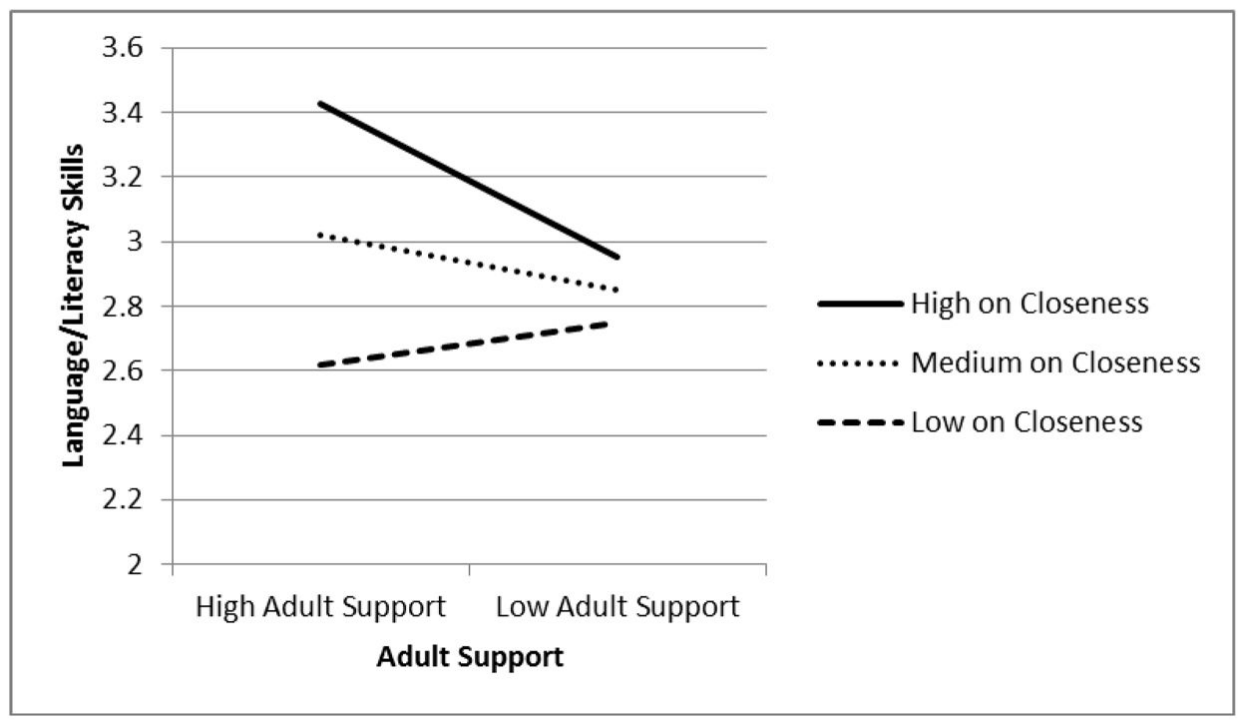
Teacher-child closeness in kindergarten as a moderator of the association between adult support and children’s language/literacy skills during the transition to kindergarten

As shown in Language and Literacy Model 2, there was a significant interaction between low adult support and teacher-reported closeness with the child (*B* = −0.03, S.E. = 0.01, *p* = .02). This interaction is depicted graphically in Figure 1, in which teacher-child closeness is shown as a moderator of the relationship between adult support and children’s language/literacy skills in kindergarten. Tests of the significance of simple slopes for low, medium, and high levels of teacher-child closeness indicated that the simple slope for high levels of closeness was the only one of the three that was significantly different from zero (*b* = −0.25*,* S.E. = 0.08, *p* = .003). As shown in Figure 1, among children who were high on closeness with the teacher, those in schools that were independently characterized as being high on adult support scored significantly higher on language/literacy in kindergarten than those in schools with lower levels of adult support. There were no significant effects of adult support on children with medium or low levels of closeness with the teacher. In short, children benefited academically from high adult support in the school only when they were close with their teacher.

The indicator for whether the teacher and child were of the same race/ethnicity became a significant predictor of children’s language/literacy skills in kindergarten in Model 2 (it was significant at the trend level in Model 1). Children who were of the same racial/ethnic background as their teacher were reported to have stronger language/literacy skills than those whose race/ethnicity differed from that of their teacher (*B* = 0.24, S.E. = 0.12, *p* = .04). No other child-level covariates emerged as significant predictors of children’s language/literacy skills in kindergarten.

#### 3.2.2 Math Skills

Math Model 1 in Table 3 shows that there was a significant main effect of low adult support, such that children in schools that were independently characterized as being low on adult support were rated by teachers as showing significantly lower math scores in kindergarten, even after controlling for their earlier math skills in Head Start (*B* = −0.22, S.E. = 0.09, *p* = .01). Three school-level covariates were also significant predictors of children’s math skills. Children in schools in which a larger proportion of students met or exceeded IL reading and math standards were rated by teachers as showing better math skills in kindergarten, net of their math skills in Head Start (*B* = 0.02, S.E. = 0.01, *p* = .04). Children in larger schools and schools with larger percentages of ELLs were rated by teachers as showing poorer math skills in kindergarten, net of their math skills in Head Start (*B* = −0.02, S.E. = 0.01, *p* = .03, and *B* = −0.02, S.E. = 0.01, *p* = .05, respectively).

Teacher-child closeness was a statistically significant predictor of children’s math skills, such that children whose kindergarten teachers reported higher levels of closeness with them were rated by teachers as having higher math scores in kindergarten (*B* = 0.05, S.E. = 0.01, *p* < .001). Again, this association between teacher-child closeness and children’s math skills in kindergarten held even after accounting for stability in children’s math skills between Head Start and kindergarten (children with better early math skills in Head Start, as measured by the NRS, were rated by teachers as showing higher math scores in kindergarten, *B* = 1.00, S.E. = 0.37, *p* = .007).

**Figure 2:**
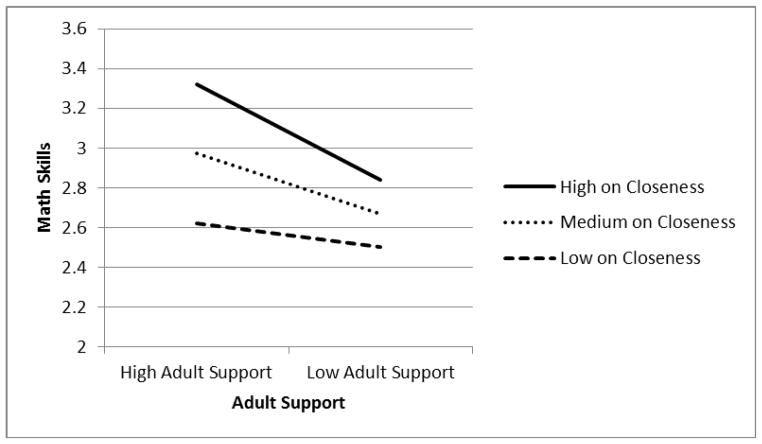
Teacher-child closeness in kindergarten as a moderator of the association between adult support and children’s math skills during the transition to kindergarten

Math Model 2 shows that there was a significant interaction between low adult support and teacher-reported closeness with the child when predicting children’s math skills (*B* = −0.02, S.E. = 0.01, *p* = .04). This interaction is depicted graphically in Figure 2, in which teacher-child closeness is shown as a moderator of the relationship between adult support and children’s math skills in kindergarten. Tests of the significance of simple slopes for low, medium, and high levels of teacher-child closeness indicated that the simple slope for high levels of closeness was the only one that was significantly different from zero (*b* = −0.27, S.E. = 0.09, *p* = .002). As shown in Figure 2, among children who were high on closeness with the teacher, those in schools that were independently characterized as being high on adult support scored significantly higher on math in kindergarten than those in schools with lower levels of adult support. The simple slope for medium levels of closeness was significant at the trend level (*b* = −0.17, S.E. = 0.10, *p* = .08). There were no significant effects of adult support on children with low levels of closeness with the teacher. As was true for children’s language and literacy skills, children’s math skills benefited from high adult support in the school only when children were close with their teacher.

## 4. Discussion

The purpose of this paper was to examine the roles of school climate and closeness in the teacher-student relationship as key predictors of low-income children’s academic skills as they made the transition to kindergarten. In service of this goal, we made use of older students’ ratings of the school environments into which children in our sample transitioned to kindergarten. By capitalizing on these school-level survey ratings of school climate, we were able to consider inputs into young children’s early academic skills from an ecologically “fresh” perspective: few previous studies of kindergartners’ academic skills have considered this dimension of children’s early educational experiences. Older students’ ratings provided an important and independent window through which to consider young children’s first school experiences.

The two dimensions of school climate examined in our analysis, unsafe climate and low adult support, reflect distinct components of the social climate in public schools in urban areas of concentrated disadvantage. An unsafe school was one in which students did not get along well, fighting was common, and students were often threatened or bullied. In schools characterized by low adult support, students felt that teachers and other adults in the school were unavailable to provide them with help and support, both in and out of the classroom. These dimensions align with two of the constructs that have emerged in the school climate literature as predictors of students’ school success: school safety and teachers’ support for students’ academic success. They also provide a valuable window into the settings that many children in urban communities such as Chicago enter at the start of the school year.

How do young children fare academically as they manage this key transition from small preschools to large urban schools at the start of their kindergarten year? Our examination of links between school climate, teacher-child closeness, and children’s academic skills as they moved into elementary school highlighted the importance of adult support at both school and classroom levels to children’s academic success. At the school level, we found main effects of low adult support on children’s language/literacy and math skills, such that children in schools with low levels of support showed poorer academic skills as they transitioned from Head Start to kindergarten. This finding is consistent with evidence that school-level measures of teachers’ commitment to students are strong predictors of students’ school performance (Brookover et al., 1978; Esposito, 1999; Hoy & Hannum, 1997). At the classroom level, we found that the nature of the teacher-child relationship was also a consistent predictor of children’s academic performance in kindergarten. Higher levels of closeness with the kindergarten teacher were significantly related to higher levels of children’s language/literacy and math skills. These findings are in line with others that suggest that higher levels of closeness with the teacher are associated with children’s academic performance in elementary school, especially among those at risk for school difficulty (Burchinal et al., 2002; Hamre & Pianta, 2001). Teacher-child closeness remained a robust predictor of children’s academic skills even after accounting for whether there was a racial/ethnic match between the child and teacher—a separate but related dimension of the teacher-child relationship.

When we probed these relationships further, we found that low adult support played a significant role in predicting children’s kindergarten academic skills in ways that were conditioned (or moderated) by the level of closeness between the student and teacher in the classroom. Specifically, higher levels of adult support (as perceived by older students attending the same school) were associated with higher levels of children’s kindergarten language/literacy and math skills for children with high levels of teacher-child closeness, even after taking into account their skills in these domains in the preschool year, but not for children with medium or low levels of teacher-child closeness. These findings suggest that children only benefited from a climate of high adult support during the transition to kindergarten if they were close to their kindergarten teacher.

Drawing on theories about the cumulative effect of risk and protective factors, low-income children seem to fare better academically when they are exposed to multiple protective factors within the school. Indeed, teacher-child closeness serves a protective role for children in schools with low levels of adult support, but children fare better when a close teacher-child relationship is coupled with high levels of adult support in the school. This suggests that there might be a multiplicative effect of exposure to both closeness in the teacher-child relationship and high levels of adult support in the school, which is consistent with evidence that children at risk for poor developmental outcomes benefit from exposure to a greater number of protective factors (Seifer et al., 1992).

Our findings provide clear evidence for the role of adult support at both school and classroom levels in supporting children’s opportunities for learning as early as kindergarten. Why might adult support play such a clear role in fostering children’s academic performance? Research with samples of older students suggests that school-wide teacher support is a key predictor of students’ sense of belonging in the school community, engagement, and motivation, and that engagement and motivation, in turn, predict student achievement (Goodenow, 1993; Goodenow & Grady, 1993; Wentzel, 1997, 1998). Findings from this work on school-level teacher support in middle school are consistent with those of studies of the dyadic teacher-child relationship in elementary school, which have also identified links between children’s early relationships with teachers, their school adaptation and engagement, and their academic success (Birch & Ladd, 1997; Pianta et al., 1995). Thus, it seems plausible that the support of teachers and other adults in the school provides children with a sense of school connectedness, which facilitates their interest and engagement in learning and their school success. Under ideal circumstances, children experience this support in both their relationship with their primary teacher and their daily interactions with adults throughout the school.

Our results are bolstered by the ways in which we controlled for children’s pre-academic skills in preschool. By including these covariates in the models we were able to at least partially control for potential confounds, such as the possibility that children with initially stronger academic skills might be enrolled in higher-quality schools. The findings highlight the close connections between students’ social and academic lives in school settings. In addition, the consistency in findings for language/literacy and math are in keeping with the high correlation between the two outcomes and suggests that the results pertain to children’s academic performance more generally rather than to language/literacy or math specifically.

In contrast to the associations found between low adult support and children’s language/literacy and math skills, we found no evidence to suggest that unsafe climate was related to children’s academic skills during the transition to kindergarten. Although some studies have found that school safety is related to older students’ academic performance (Bowen & Bowen, 1999), our results with younger children entering kindergarten did not yield similar findings. There is evidence that school attendance may mediate the association between school safety and elementary-school students’ academic achievement (Chen, 2007). If this is the case, then links between unsafe climate and children’s academic skills might not be apparent immediately but might emerge over the longer term (Walters & Bowen, 1997). Since our measures of children’s academic skills were administered in winter of the kindergarten year, only a few months after children began kindergarten and entered elementary school, there may not have been enough time to see associations emerge between unsafe climate and children’s academic skills, especially if younger children are less likely to be exposed to the school safety problems reported by older students. This might be the case if younger children are more sheltered from safety problems or have little interaction with older students, who might be more likely to engage in violence and bullying. Future research should examine whether links between school safety and children’s academic skills in elementary school are more likely to emerge over longer periods of time.

At the child level, in addition to finding associations between teacher-child closeness and children’s academic skills during the transition to kindergarten, we found that direct assessments of children’s pre-academic skills in spring of the Head Start year were consistent predictors of children’s academic performance in winter of the kindergarten year. Early letter-naming and receptive-vocabulary skills were strong predictors of children’s language/literacy skills in kindergarten and early math skills were strong predictors of children’s kindergarten math skills. These results support other evidence that children’s early academic skills are key predictors of later academic achievement (Mullis & Jenkins, 1990). Whether the teacher and child were of the same race/ethnicity emerged as a significant predictor of children’s language/literacy skills in the model that included interactions between teacher-child closeness and school climate. Children who were of the same racial/ethnic background as their teacher had stronger teacher-reported language/literacy skills than those who were of a different race/ethnicity. This finding is in line with previous evidence that a racial/ethnic mismatch between teacher and student is associated with poorer student-teacher relations and poorer student academic outcomes (Dee, 2004; Zimmerman, Khoury, Vega, Gil, & Warheit, 1995), and suggests that a racial/ethnic match may be particularly important for low-income, racially and ethnically diverse children.

Three school-level covariates emerged as significant predictors of children’s academic skills during the transition to kindergarten. Children in larger schools showed poorer academic skills in kindergarten, as did children in schools with larger percentages of ELLs and those in schools in which a smaller percentage of students were proficient in math and reading (when predicting math skills only). These results suggest that both the size and composition of the student body is associated with children’s academic skills, even after accounting for children’s earlier pre-academic skills in the Head Start year. The findings are consistent with large bodies of research that suggest that smaller schools offer better learning environments for children (Fowler & Walberg, 1991; Lee & Loeb, 2000) and that peer effects, including higher overall academic performance in the school, are associated with elementary-school children’s individual achievement scores (Aikens & Barbarin, 2008; Han & Bridglall, 2009).

There are a number of limitations to this study. First, our analysis of the relationships between school climate and children’s academic skills was correlational in nature, so we cannot draw any causal inferences from our results. Although it seems likely that exposure to an elementary school climate characterized by high levels of adult support leads to improvements in children’s academic skills, it is also plausible that children with stronger academic skills select into schools with higher levels of adult support (if, for example, their parents are more resourceful or more motivated to enroll them in good schools). We controlled for a number of child, family, and school characteristics in our models to address the threat of selection bias, including children’s pre-academic skills in Head Start, but were unable to account for all sources of omitted variable bias.

Second, we relied on teacher-reported measures of children’s closeness with the teacher and academic skills in kindergarten. As a result, our dependent variables may have suffered from individual teacher biases and the associations between kindergarten teachers’ reports of teacher-child closeness and children’s academic skills may have suffered from the challenges associated with shared method variance. In light of this, having a more objective measure of children’s academic skills would have been desirable. It is important to note, however, that the strong associations found between the direct assessments of children’s pre-academic skills in Head Start and teacher-reported measures of children’s academic skills in kindergarten, as well as the associations between CPS DIBELS scores and teacher reports of children’s language/literacy skills, provide evidence of the validity of our dependent measures. Furthermore, the inclusion of controls for children’s pre-academic skills in preschool made our models—even those that relied largely on teacher reports—conservatively specified. Finally, we controlled for a well-established source of teacher-report bias, that of teacher-child match versus mismatch on racial/ethnic status. Our findings held up well under that increased level of empirical scrutiny.

Third, we had the opportunity to capitalize on a new city-wide survey of middle-school students’ reports of school climate, which served as both a limitation and strength of the analysis. It was a limitation in that we do not know the extent to which middle-school students’ perceptions of school climate reflected the experiences of the elementary-school children in the same school. On the other hand, using middle-school students’ ratings of school climate may have been preferable to using the school-climate ratings of the elementary-school children in our sample. Middle-school students’ ratings are independent of any biases related to elementary-school children’s school experiences and academic performance. Middle school students are also likely to provide more reliable ratings of school climate than kindergarten students, resulting in less “noisy” measures.

With these caveats in mind, this paper offers several significant contributions to the fields of applied developmental and educational research. We examined two dimensions of the social climate in urban schools in low-income communities. Little prior research has focused on school climate in largely low-income schools. In addition, we considered *school-level* predictors of changes in young, low-income children’s academic skills across a key educational transition. Prior research on the role of school climate in shaping children’s academic skills has focused largely on older students in middle and high schools, and no research that we are aware of on elementary school climate has focused on children in kindergarten. Research on younger children has focused primarily on *classroom* rather than school characteristics. In this analysis we examined both school- and classroom-level components of children’s school experiences as they entered formal schooling. Our findings offer new perspectives on how to understand low-income children’s entry into large and sometimes bewildering settings, as they leave smaller community- and neighborhood-based preschools for the wider world of school. Specifically, they highlight the important role that adult support plays at both school and classroom levels in fostering young children’s success in school. These findings contribute to an emerging area of interest in understanding the ways in which elementary-school settings can support children’s early academic success.

## Figures and Tables

**Table 1:** Descriptive statistics for variables used in multilevel models

Variable	*M*	*SD*	Range
Outcome variables (all measured at kindergarten)			
Language and Literacy (n = 241)	2.89	1.06	1–5
Math (n = 220)	2.82	1.06	1–5

Child and family characteristics (*n* = 242)			
Child’s race/ethnicity			
White/non-Hispanic	0.05	0.21	0–1
African American	0.62	0.49	0–1
Hispanic	0.30	0.46	0–1
Bi-racial	0.03	0.18	0–1
Child is male	0.52	0.50	0–1
Child’s age at kindergarten (years)	5.25	0.50	4–7
Teacher-child closeness in kindergarten	33.05	5.75	16–40
Family income-to-needs ratio in Head Start	0.69	0.57	0–3.57
Child’s home language is Spanish	0.15	0.36	0–1
Teacher and child are of same race/ethnicity	0.57	0.50	0–1
Early math skills in Head Start	0.59	0.17	0.16–0.95
Letter naming skills in Head Start	0.54	0.38	0–1
Receptive vocabulary skills in Head Start	0.61	0.17	0.13–0.96

School characteristics (*n* = 102)			
School size (total enrollment)	743.04	684.36	157–6,306
Percentage of low-income students in the school	89.29	13.99	12–100
Percentage of English Language Learners (ELLs) in the school	14.74	18.26	0–55
Percentage of students in the school who met/exceeded state reading and math standards	65.40	14.48	35.20–97.70
Unsafe climate	0.17	0.84	−0.85–1.64
Low adult support	−0.21	0.88	−3.95–1.33

**Table 2:** Correlations among level-1 variables included in multilevel models

	2.	3.	4.	5.	6.	7.	8.	9.	10.	11.	12.
1. Child’s race/ethnicity is African American	0.04	0.06	−0.06	−0.05	−0.53***	0.04	0.29***	0.01	0.12†	−0.03	−0.08
2. Child is male	1	−0.07	−0.16	0.08	−0.13*	−0.11	−0.13†	−0.17**	−0.13†	−0.12†	−0.13†
3. Child’s age at kindergarten (years)		1	0.09	−0.05	0.04	−0.10	0.07	0.21**	0.22**	0.14*	0.08
4. Teacher-child closeness in kindergarten			1	0.15*	−0.02	0.08	0.10	0.29***	0.25***	0.38***	0.35***
5. Family income-to-needs ratio in Head Start				1	0.02	0.05	0.11	−0.04	0.10	0.17**	0.14*
6. Child’s home language is Spanish					1	0.14*	−0.22***	−0.04	−0.13*	−0.07	0.01
7. Teacher and child are of same race/ethnicity						1	−0.05	0.01	−0.16*	−0.03	−0.03
8. Letter naming (Head Start)							1	0.27***	0.56***	0.33***	0.30***
9. Receptive vocabulary (Head Start)								1	0.58***	0.34***	0.24***
10. Early math (Head Start)									1	0.44***	0.35***
11. Language and Literacy (K)										1	0.88***
12. Math (K)											1

Note.

† *p* < .10,

* *p* < .05,

** *p* < .01,

*** *p* < .001.

**Table 3:** Parameter coefficients (and standard errors) from multilevel models examining child- and school-level predictors of children’s academic skills in kindergarten

Variable	Language and Literacy				Math			

	Model 1		Model 2		Model 1		Model 2	

	*B*	(S.E.)	*B*	(S.E.)	*B*	(S.E.)	*B*	(S.E.)
Child’s race/ethnicity is African American	−0.13	(0.24)	−0.17	(0.25)	0.11	(0.28)	0.06	(0.30)
Child is male	−0.04	(0.12)	−0.03	(0.12)	−0.13	(0.10)	−0.11	(0.09)
Child’s age at kindergarten (years)	0.16	(0.13)	0.17	(0.14)	0.02	(0.15)	0.03	(0.15)
Teacher-child closeness in kindergarten	0.04***	(0.01)	0.04***	(0.01)	0.05***	(0.01)	0.05***	(0.01)
Family income-to-needs ratio in Head Start	0.12	(0.10)	0.13	(0.09)	0.06	(0.09)	0.07	(0.09)
Child’s home language is Spanish	−0.10	(0.25)	−0.10	(0.25)	0.16	(0.25)	0.13	(0.26)
Teacher and child are of same race/ethnicity	0.23†	(0.12)	0.24*	(0.12)	0.25	(0.18)	0.23	(0.18)
Letter naming (Head Start)	0.77***	(0.19)	0.79***	(0.19)				
Receptive vocabulary (Head Start)	0.86*	(0.41)	0.84*	(0.40)				
Early math (Head Start)					1.00**	(0.37)	0.98**	(0.36)

% of low-income students in the school	−0.14	(0.72)	−0.17	(0.74)	0.03	(0.67)	0.03	(0.68)
% of students in the school who met/exceeded state reading and math standards	0.01	(0.01)	0.01	(0.01)	0.02*	(0.01)	0.02*	(0.01)
Number of children enrolled in the school	−0.02*	(0.01)	−0.02*	(0.01)	−0.02*	(0.01)	−0.02*	(0.01)
% of English language learners (ELLs) in the school	−0.02*	(0.01)	−0.02*	(0.01)	−0.02*	(0.01)	−0.02*	(0.01)
Unsafe climate	−0.09	(0.12)	−0.12	(0.12)	0.03	(0.16)	−0.01	(0.16)
Low adult support	−0.16*	(0.08)	−0.10	(0.08)	−0.22**	(0.09)	−0.17†	(0.10)
Unsafe climate X Teacher-child closeness in kindergarten			0.00	(0.01)			0.01	(0.01)
Low adult support X Teacher-child closeness in kindergarten			−0.03*	(0.01)			−0.02*	(0.01)

Constant	1.08	(1.22)	1.12	(1.24)	0.62	(1.34)	0.72	(1.37)

Note.

† *p* < .10,

* *p* < .05,

** *p* < .01,

*** *p* < .001;

Level-1 equation: Academic skills in kindergarten = B_0j_ + B_1j_*(Child’s race/ethnicity) + B_2j_*(Child is male) + B_3j_*(Child’s age) + B_4j_*(Teacher-child closeness in kindergarten) + B_5j_*(Family income-to-needs ratio) + B_6j_*(Child’s home language is Spanish) + B_7j_*(Teacher and child are of same race/ethnicity) + B_8j_*(Child’s pre-academic skills in Head Start) + r_ij_. Level-2 equation: B_0j_ = G_00_ + G_01_*(School size) + G_02_*(% low-income students in school) + G_03_*(% ELL students in school) + G_04_*(% students in school who met/exceeded state reading/math standards) + G_05_*(Unsafe climate) + G_06_*(Low adult support) + G_07_*(Unsafe climate X Teacher-child closeness in kindergarten) + G_08_*(Low adult support X Teacher-child closeness in kindergarten) + u_0j_.

## Footnotes

Note 1 The CSRP intervention was implemented with two cohorts of children, with Cohort 1 participating (at time T) in 2004–2005 and Cohort 2 participating (at time T) in 2005–2006. Cohorts 1 and 2 were in kindergarten (at time T+1) in 2005–2006 and 2006–2007, respectively.

Note 2 Because of high levels of missing data on the kindergarten survey (44%), the family’s income-to-needs ratio from the Head Start year (T) was used in the analysis instead of the equivalent measure from the kindergarten year (T+1). The set of site-pair dummies captures observed and unobserved heterogeneity among children enrolled in CSRP Head Start sites with differing resources and across different neighborhoods (see Raver et al., 2009, for more information). Because the variable for cohort (1 versus 2) was perfectly correlated with the set of site-pair dummies, we did not control for cohort in the analysis.

Note 3 Two of these covariates (school enrollment and the percentage of low-income students in the school) were rescaled by dividing the raw variables by 100 in order to make the parameter estimates more easily interpretable.
